# Whole-genome sequence data and analysis of type strains ‘*Pusillimonas nitritireducens’* and ‘*Pusillimonas subterraneus’* isolated from nitrate- and radionuclide-contaminated groundwater in Russia

**DOI:** 10.1016/j.dib.2018.10.060

**Published:** 2018-10-25

**Authors:** Denis S. Grouzdev, Tatiyana P. Tourova, Tamara L. Babich, Margarita A. Shevchenko, Diyana S. Sokolova, Ruslan R. Abdullin, Andrey B. Poltaraus, Stepan V. Toshchakov, Tamara N. Nazina

**Affiliations:** a Institute of Bioengineering, Research Center of Biotechnology, Russian Academy of Sciences, Moscow, Russian Federation; b Winogradsky Institute of Microbiology, Research Center of Biotechnology, Russian Academy of Sciences, Moscow, Russian Federation; c Immanuel Kant Baltic Federal University, Kaliningrad, Russian Federation; d Engelhardt Institute of Molecular Biology, Russian Academy of Sciences, Moscow, Russian Federation; e V.I. Vernadsky Institute of Geochemistry and Analytical Chemistry of Russian Academу of Sciences, Moscow, Russian Federation

**Keywords:** Draft genome, Gene prediction, *‘Pusillimonas nitritireducens’*, ‘*Pusillimonas subterraneus’*, denitrification, Metal resistance

## Abstract

Two strains, ‘*Pusillimonas nitritireducens’* JR1/69-2-13^T^ and ‘*Pusillimonas subterraneus’* JR1/69-3-13^T^, of aerobic, motile, Gram-negative, non-spore-forming, organotrophic, psychrotolerant bacteria were isolated from a sample of nitrate- and radionuclide-contaminated groundwater in Russia. Here we describe the draft genomes of these strains. The sequenced and annotated genome of the strain JR1/69-2-13^T^ contained 4.3 Mbp with 4108 protein-coding genes. The genome of the strain JR1/69-3-13^T^ contained 4.5 Mbp with 4260 protein-coding genes. Genome analysis of both strains provides an insight into the genomic basis of their resistance to nitrate, heavy metals and metalloids. The draft genome sequences of strains ‘*Pusillimonas nitritireducens’* JR1/69-2-13^T^ and ‘*Pusillimonas subterraneus’* JR1/69-3-13^T^ are available at DDBJ/EMBL/GenBank under the accession nos. https://www.ncbi.nlm.nih.gov/nuccore/PDNV00000000 and https://www.ncbi.nlm.nih.gov/nuccore/PDNW00000000, respectively.

**Specifications table**

**Table:** 

Subject area	Biology
More specific subject area	Microbiology and genomics.
Type of data	Genome sequencing data, table, text file, image and figure.
How data was acquired	Transmission electron microscope (JEOL JEM-1010, Japan), Shotgun draft genome DNA sequencing using MiSeq platform (Illumina) and bioinformatics applications.
Data format	Draft genome sequences and gene prediction.
Experimental factors	Isolation and characterization of strains JR1/69-2-13^T^ and JR1/69-3-13^T^. Genomic DNA extraction and sequencing procedure.
Experimental features	Draft genome sequencing was performed according to Illumina sequencing protocols for DNA-seq followed by annotation and gene description.
Data source location	Strains were isolated from nitrate- and radionuclide-contaminated groundwater sample (Ozyorsk town, South Urals, Russia). Latitude: 55°38′ N and Longitude: 60°47′ E.
Data accessibility	The draft genome sequences of strains JR1/69-2-13^T^ and JR1/69-3-13^T^ are available at DDBJ/EMBL/GenBank under the accession nos. PDNV00000000 and PDNW00000000, respectively. (https://www.ncbi.nlm.nih.gov/nuccore/PDNV00000000, https://www.ncbi.nlm.nih.gov/nuccore/PDNW00000000)

**Value of the data**

• Draft genome sequences of ‘*P. nitritireducens’* and ‘*P. subterraneus’* will create an opportunity for comparative taxonomic studies of the closely related *Pusillimonas* – *Candidimonas* group.
• Draft genomes and identified genes of new strains will provide insights into the molecular mechanisms by which these strains transform nitrate or nitrite to dinitrogen gas and survive in the nitrate- and radionuclides-contaminated environment.
• This data set will be useful for the scientific community, working in the area of functional and phylogenetic diversity of microorganisms in environments contaminated with radioactive waste, and for development of biotechnologies for *in situ* bioremediation of groundwater by nitrate removal.

## 1 Data

**Fig. 1:**
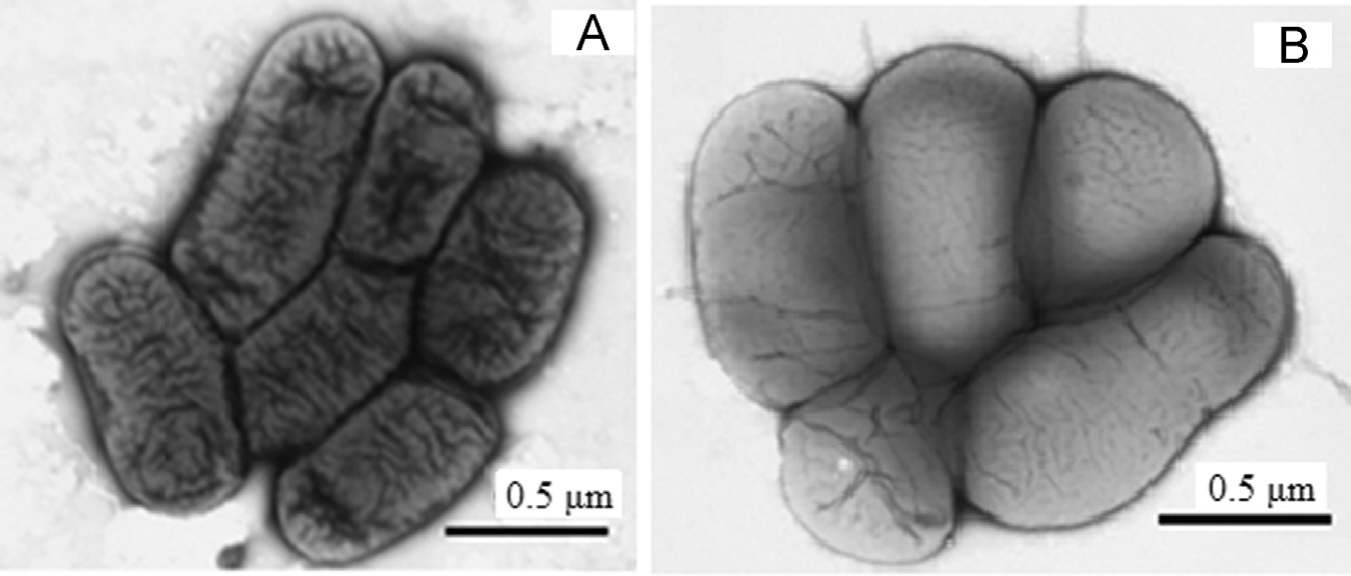
Transmission electron micrographs of *‘Pusillimonas nitritireducens’* JR1/69-2-13^T^ (A) and *Pusillimonas subterraneus’* JR1/69-3-13^T^ (B). Scale bars are 0.5 μm.

**Fig. 2:**
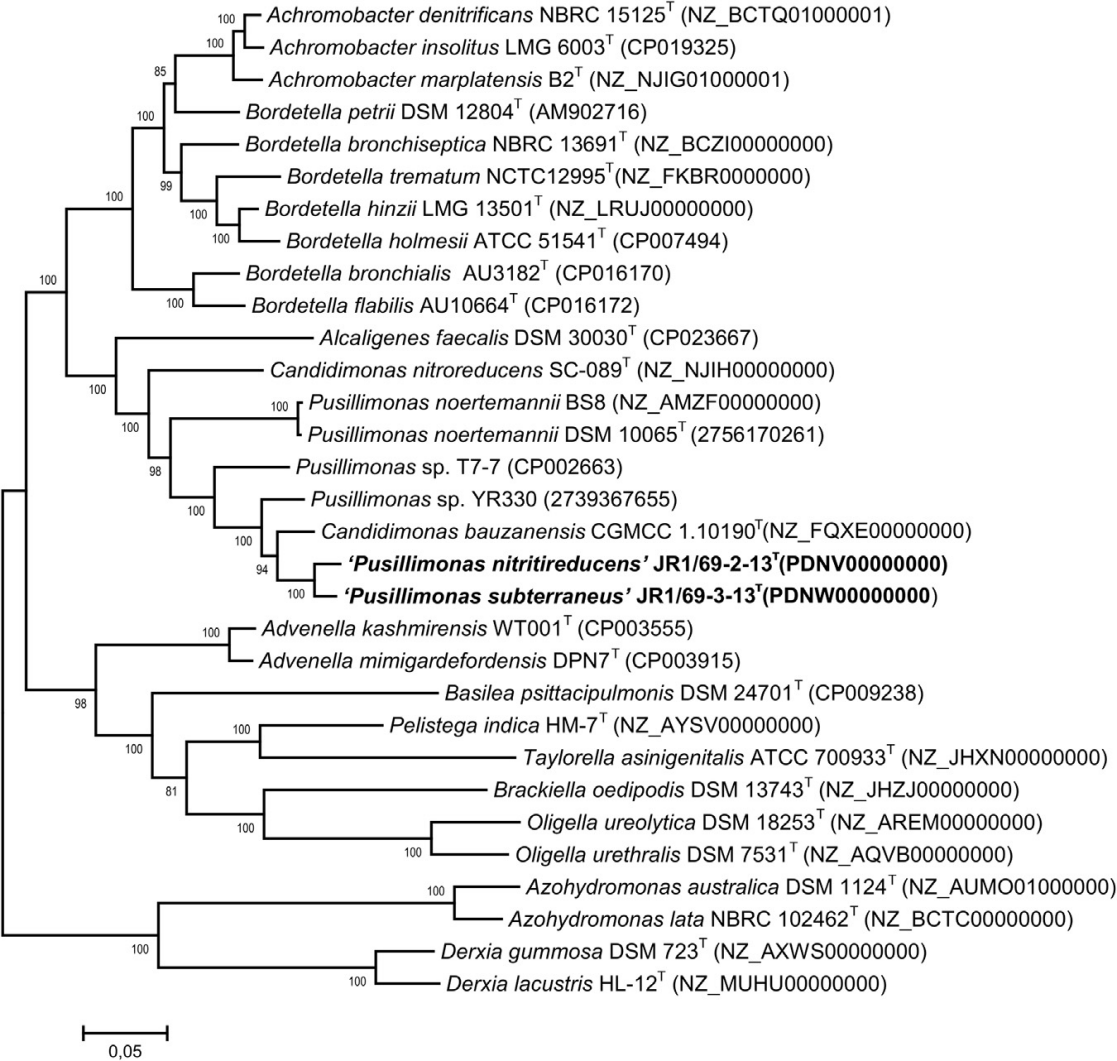
Phylogenetic tree inferred from the comparison of 492 concatenated *Alcaligenaceae* core protein-coding genes (167141 aa) showing the position of strains JR1/69-2-13^T^ and JR1/69-3-13^T^. Bootstrap values are indicated at branch nodes.

In the present work, we describe the draft genome sequences and gene prediction of two strains of aerobic organotrophic bacteria, JR1/69-2-13^T^ and JR1/69-3-13^T^ (Fig. 1), isolated from nitrate- and radionuclides-contaminated groundwater collected near the industrial reservoir for liquid radioactive waste (Ozyorsk town, South Urals, Russia) [1]. Functional and phylogenetic diversity of the microorganisms inhabiting the subterranean water-bearing horizons associated with the plants processing uranium and other radionuclides remain insufficiently studied [2], [3]. Two strains, JR1/69-2-13^T^ and JR1/69-3-13^T^, were chosen for genome sequencing with the goal of determination of their taxonomic position and identification of the genetic determinants providing for their occurrence in the environment contaminated with radioactive waste. Based on the 16S rRNA gene phylogeny data, both strains were found to belong to the domain *Bacteria*, the phylum *Proteobacteria*, the class *Betaproteobacteria*, the order *Burkholderiales*, the family *Alcaligenaceae* [4], [5], [6], [7], [8]. The 98.7% similarity level between the 16S rRNA gene sequences of these two strains indicated that they could probably belong to different species. The 16S rRNA gene sequences of the strains JR1/69-2-13^T^ and JR1/69-3-13^T^ were phylogenetically closely related to those of the species of the genera *Pusillimonas* and *Candidimonas* [9], [10], [11], [12], [13], [14], [15]. As was demonstrated in previous studies, differentiation between the genera of the family *Alcaligenaceae* on the basis of their phenotypic characteristics is not possible [9], [10], [14], [16]. Genomic features of strains *Pusillimonas nitritireducens’* JR1/69-2-13^T^ and ‘*Pusillimonas subterraneus’* JR1/69-3-13^T^ are presented in Table 1. Numerous genes responsible for heavy metal tolerance and detoxification were identified in their genomes. In the JR1/69-3-13^T^ genome, the denitrification gene cluster included the genes predicted to encode the enzymes for sequential reduction of nitrate to dinitrogen gas, which was consistent with the complete denitrification phenotype of the strain. In the JR1/69-2-13^T^ genome, the genes encoding nitrite reduction to N_2_ were revealed. *In silico* DNA-DNA hybridization (dDDH) values against reference genomes of *Pusillimonas* and *Candidimonas* strains were within the range of 19.3–28.8, and were below the 70% threshold to differentiate bacterial species [17]. These data and position of strains JR1/69-2-13^T^ and JR1/69-3-13^T^ on the tree of 492 concatenated core protein-coding genes (Fig. 2) testify to their belonging to two novel species within the *Pusillimonas* genus with proposed names ‘*Pusillimonas nitritireducens’* and ‘*Pusillimonas subterraneus’*, respectively. The draft genome sequences of strains JR1/69-2-13^T^ and JR1/69-3-13^T^ are deposited in DDBJ/EMBL/Genbank under the accession nos. PDNV00000000 and PDNW00000000, respectively. The versions described in this paper are the first versions of the genome of each strain.

**Table 1:** Genomes features of ‘*Pusillimonas nitritireducens*’ JR1/69-2-13^T^ and ‘*Pusillimonas subterraneus’* JR1/69-3-13^T^.

Features	JR1/69-2-13^T^		JR1/69-3-13^T^	
	Value	% of Total	Value	% of Total
Genome size (bp)	4,310,404	100.00	4,544,755	100.00
DNA coding (bp)	3,920,667	90.96	4,139,718	91.09
DNA G+C (bp)	2,467,295	57.24	2,632,477	57.92
DNA scaffolds	38	100.00	68	100.00
Total genes	4122	100.00	4270	100.00
Protein coding genes	3981	96.58	4118	96.44
RNA genes	51	1.24	54	1.26
Pseudo genes	90	2.18	98	2.30
Genes in internal clusters	1312	31.46	1321	30.59
Genes with function prediction	3547	85.04	3686	85.34
Genes assigned to COGs	3194	76.58	3286	76.08
Genes with Pfam domains	3626	86.93	3778	87.47
Genes with signal peptides	431	10.33	447	10.35
Genes with transmembrane helices	997	23.90	1044	24.17
CRISPR repeats	–		–

## 2 Experimental design, materials, and methods

### 2.1 Isolation of the strains JR1/69-2-13^T^ and JR1/69-2-13^T^

Strains JR1/69-2-13^T^ and JR1/69-2-13^T^ were isolated from nitrate- and radionuclide-contaminated groundwater sample collected from the depth of 44 m at a distance 3.2 km from the Karachai Lake (Ozyorsk town, South Urals, Russia) [1], [18]. Two strains were purified by successive transfer from the liquid TEG medium containing bacto-trypton (5.0 g L^−1^), yeast extract (2.5 g L^−1^), glucose (1.0 g L^−1^), and distilled water (1 L, pH 7.0) to solid TEG medium with agar-agar (15.0 g L^−1^). Bacteria were incubated at 22–30 °C. Strains JR1/69-2-13^T^ and JR1/69-3-13^T^ were deposited in the Russian Collection of Microorganisms as VKM В-3222^T^ and VKM В-3223^T^, respectively.

### 2.2 DNA isolation and sequencing

Strains JR1/69-2-13^T^ and JR1/69-3-13^T^ were grown in TEG liquid medium at 30 °C for 72 h. The cells were harvested by centrifugation. The cell integrity was accessed by transmission electron microscopy (JEOL JEM-1010, Japan) of the cells negatively stained with 1% phosphotungstic acid (Fig. 1A and B). DNA of each strain was isolated from the biomass by the phenol-chloroform-based method as described previously [19]. NEBNext Ultra DNA library preparation kit (New England Biolabs, USA) was used to prepare fragment libraries for genome sequencing. Next-generation shotgun-sequencing was performed on MiSeq (Illumina Inc., USA) at the Immanuel Kant Baltic Federal University (Kaliningrad, Russian Federation).

### 2.3 Genome assembly

Raw sequence reads were subjected to stringent quality filtering with CLC Genomics Workbench 10.0 (Qiagen, Germany). After filtering, sequencing adapters were trimmed with SeqPrep tool (https://github.com/jstjohn/SeqPrep). Finally, 2,802,807 and 2,451,909 read pairs were used for *de novo* assembly of JR1/69-2-13^T^ and JR1/69-3-13^T^ genomes, respectively. The reads were assembled with SPAdes 3.10.0 [20] and refined by length and coverage using CLC Genomics Workbench 10.0 software (Qiagen, Germany).

### 2.4 Genome annotation

Identification of protein-coding sequences and primary annotation was performed using the NCBI Prokaryotic Genome Automatic Annotation Pipeline (PGAAP) [21]. Additional gene prediction and functional annotation were performed in the Rapid Annotation using Subsystems Technology (RAST) server [22] and Integrated Microbial Genome-Expert Review pipeline [23], respectively. Analysis of metal resistance was performed with the Antibacterial Biocide and Metal Resistance Genes Database (BacMet) [24]. *In silico* DNA-DNA hybridization (dDDH) was carried out with the online genome-to-genome calculator (GGDC 2.0) provided by the DSMZ [17].
