# Supplementary material for: Whole-genome sequence data and analysis of type strains ‘Pusillimonas nitritireducens’ and ‘Pusillimonas subterraneus’ isolated from nitrate- and radionuclide-contaminated groundwater in Russia
Source: Data Brief. 2018 Oct 25;21:882–7. doi: 10.1016/j.dib.2018.10.060 (PMC6222257; doi:10.1016/j.dib.2018.10.060)
Supplement: Supplementary file 1 — Transparency document. [file mmc1.pdf]

# Conflict of Interest Form

We wish to confirm that there are no known conflicts of interest associated with this publication. We confirm that the manuscript has been read and approved by all named authors and that there are no other persons who satisfied the criteria for authorship but are not listed. We further confirm that the order of authors listed in the manuscript has been approved by all of us.

Dr. Tamara N. Nazina
